# Neck of femur fractures treated with the femoral neck system: outcomes of one hundred and two patients and literature review

**DOI:** 10.1007/s00264-022-05414-0

**Published:** 2022-05-11

**Authors:** Amit Davidson, Shlomo Blum, Elad Harats, Erick Kachko, Ahmad Essa, Ram Efraty, Amos Peyser, Peter V. Giannoudis

**Affiliations:** 1 Academic Department of Trauma and Orthopaedic Surgery, School of Medicine, University of Leeds, Leeds, UK; 2 NIHR Leeds Biomedical Research Center, Chapel Allerton Hospital, Leeds, UK; 3 Department of Orthopaedic Surgery, Shaare Zedek Medical Center, Jerusalem, Israel; 4 Department of Orthopaedic Surgery, Shamir (Assaf Harofeh) Medical Center, Zerifin, Israel

**Keywords:** Femoral neck fractures, FNS, Femoral neck system, Revision rate, Complications, Risk factors

## Abstract

**Introduction:**

The recently developed femoral neck system (FNS) for treatment of femoral neck fractures (FNF), comprises theoretical biomechanical advantages compared to other implants. The aim of this study was to validate the safety and to report outcomes of patients treated with the FNS.

**Method:**

A retrospective multicentric analysis of patients treated by FNS with a minimum of three months of follow-up. Details analysed from three medical centres were operative duration, estimated blood loss, initial hospitalisation duration, fixation quality as well as complications and reoperation rate. Patients who had revision surgery were compared to all other patients to identify risk factors for failure. In addition, a literature review was performed to analyse data on FNS clinical implementation and patient’s outcomes. The two data sets were combined and analysed.

**Results:**

One-hundred and two patients were included in this study cohort with an average follow-up of seven months (range 3–27). Ten papers were included in the literature review, reporting data on 278 patients. Overall, 380 patients were analysed. Average age was 62.6 years, 52% of the fractures were classified as Gardens 1–2. Overall, the revision rate was 9.2% (14 patients diagnosed with cut-out of implant, 10 with AVN, 8 with non-union and 8 with hardware removal). For the 102 patients in the cohort risk factors for reoperation included patients age, surgeon seniority and inadequate placement of the implant.

**Conclusion:**

This study shows that FNS is a safe treatment option for FNF. Intra-operative parameters and failure rates are comparable to previously reported rates for this implant and other frequently used implants.

## Introduction

Femoral neck fractures (FNF) are common and account for over 50% of all hip fractures [1]. Standard treatment for FNF is surgical. The surgical treatment can be either internal fixation or arthroplasty, depending on bone quality, fracture severity and patient’s age [2]. Over the past decades, great effort has been put into deciphering specific biomechanical characteristics of FNF to develop an optimised fixation construct [3]. The recently developed femoral neck system (FNS), (DePuy Synthes, Raynham, MA, USA) comprises the theoretical mechanical advantages of combining compression and anti-rotation qualities during internal fixation. The effective solution the FNS design provides, involves the screw-plate construct, allowing stronger fixation, as well as a combination of blade and anti-rotation screw, that improves axial and rotational stability. Biomechanical studies have shown axial and rotational stability superiority of FNS implant over the traditional cannulated screws (CS) and the dynamic hip screw (DHS) [4, 5]. In addition, the FNS is relatively a minimally invasive procedure which theoretically reduces blood loss and infection risk.

There are only few reports discussing the clinical implementation of FNS [6–15]. Studies are retrospective in nature and include small number of patients. Surgical workflow and surgeons experience operating with this implant were not evaluated. Moreover, intraoperative parameters such as duration of surgery and blood loss have not been fully explored. In addition, medium- and long-term patient’s outcomes, with relation to re-operation, mechanical failure and avascular necrosis (AVN) rates, are inconclusive.

While the FNS has mechanical superiority over the traditional implants in cadaveric experiments, it is crucial to evaluate the safety and report outcomes of this new implant.

The aim of this study therefore was to investigate the outcome of patients with FNF managed with FNS in our institutions and in the published literature. Moreover, we wish to validate the safety of this implant, and compare the outcome of patients treated with FNS to other implants. Our hypothesis was that the FNS implant would demonstrate if not better, analogous results to similar implants used for fixation of FNFs.

More specifically, the study’s objectives were as follows:

1. Analyse data obtained from three medical centres on patient treated by FNS regarding the operative duration, estimated blood loss and initial hospitalisation duration.
2. Assess patients’ medium- and long-term outcomes regarding AVN, mechanical failure, re-operation rates and the associated risk factors.
3. Perform literature review and analyse data reported on FNS clinical implementation and patient’s outcomes.
4. Compare the data collected on FNS to other implants published outcome and peri-operative parameters.

## Patients and methods

Data on FNF patients treated with FNS from three different medical centres, between 01 March 2019 and 01 August 2021, was retrospectively collected and analysed. Institutional review board approval, from all institutes participating in the study, was obtained. Inclusion criteria for the study were patients with FNF treated with FNS and a minimum of three month post-operative follow-up. Exclusion criteria were open fractures, evidence of pathological fracture, skeletally immature patients and revision procedure for a failed fixation. Data was collected from electronic records of each institution and included patients’ demographics and recorded comorbidities. Comorbidities were defined as six distinct categories: cardiac, neurological, chronic kidney disease, chronic lung disease, diabetes mellitus and active malignancy. Data on the initial hospitalisation was collected regarding time from admission to surgery and length of hospital stay. In addition, operation time, estimated operative blood loss, which was estimated by the surgeon at the end of the procedure and surgeon seniority, qualified orthopaedic surgeon versus a trainee were recorded. Quality of reduction was evaluated by radiographic measurements performed on initial postoperative radiographs. Measurement of the tip apex distance (TAD) [16] and Parker ratio [17] in the anterior posterior and lateral radiographs was obtained. The rationale to add the Parkers ratio was that this measurement evaluates the importance of the position of the blade in the neck and not only the position of the tip of the blade.

Fracture classification and the radiographic measurements were performed by two of the authors. Fractures were classified according to the Garden classification [18]. Fractures were considered stable if classified as types 1 and 2 and unstable for types 3 or 4.

Treatment outcome for all patient was evaluated by collecting data from the follow-up records on, follow-up duration, reports of mechanical failure of implant (cut-out), nonunion (defined as no sign of union six months after surgery) and re-operations. Re-operated patients were evaluated for the diagnostic reason for the reoperation, time interval from primary surgery and the secondary procedure performed. Patients in the cohort were divided into two groups: group (1) underwent re-operation and group (2) did not require additional surgical procedure. Groups were compared for patients’ factors (demographics and comorbidities), fracture classification and surgical procedure-related collected data in order to evaluate specific risk factors for re-operation.

Data was collected from centres which had different implants for treatment of FNF. At one centre, FNS was the only construct used for FNF fixation, whereas at the other two centres either FNS, DHS or CS were applied for internal FNF fixation. The decision which implant to use was taken by the senior surgeon. Generally, elderly patients (aged above 65) suffering from a displaced fracture (Gardens 3–4), were treated by arthroplasty, whereas younger patients were treated by internal fixation, regardless to the displacement rate of the fracture, in all centres.

### Systematic review of the literature

The medical search engine employed in this study was PubMed; medical subject headings words used included FNS, femoral neck system and femur fractures. Articles reporting on FNS clinical outcomes were collected and analysed. Inclusion criteria were papers reporting on patients treated with FNS for FNF which were published in English. Case reports, biomechanical studies and non-English publications were excluded. When available, data obtained in the literature review was collected for the variables described above.

For the statistical analysis of this study, contingency data were analysed using two-tailed *χ*^2^ test or Fisher exact test, as appropriate. Continuous variables were compared using the Student’s *t* test.

In the systematic literature review section, literature mean numbers were calculated as weighted means, according to each sample size proportion out of all.

## Results

One hundred twenty-five patients treated with FNS were collected from three medical centres. However, twenty-three were excluded as they did not have at least three months of post-operative follow-up. In total, 102 patients (53 male) with a mean age of 62.9 years were included in this study. Data on demographics, fracture classification, surgical time, estimated blood loss, initial hospitalisation duration and patient comorbidities is presented in Table 1. The average follow-up was seven months (range 3 to 27). Nine patients underwent revision surgery, out of which five were due to a failure of fixation and cut-out of the blade, three due to AVN and one as a result of fracture nonunion. All patients who required re-operation were treated by hip arthroplasty. The average time interval from the initial surgery to the revision surgery was 18 weeks, ranging from two to 36 weeks. Two patients were diagnosed with AVN by magnetic resonance imaging (MRI) but did not require surgical treatment as they were diagnosed with low-grade AVN stage and had minor clinical symptoms at their last follow-up examination. One patient, 93-year-old male, was diagnosed with mechanical failure of the implant (cut-out) six weeks post- operation. This patient was bedridden before the operation and suffered from major comorbidities, therefore he was treated non-operatively.

**Table 1:** Description of demographics, fracture classification, perioperative characteristics and reoperations cause, for the study cohort and literature review

	Study cohort	Literature	Overall
Number of patients	102	278	380
Average age [years] (SD)	62.9 (16.5)	64.7^*^ (16)	62.6^*^ (16.15)
Gender			
Male	53/102 (52%)	136/278 (49%)	189/380 (50%)
Female	49/102 (48%)	142/278 (51%)	191/380 (50%)
Fracture classification			
Gardens 1–2	75/102 (73%)	122/278 (45%)	197/380 (52%)
Gardens 2–4	27/102 (27%)	156/278 (55%)	127/380 (48%)
Average hospitalising duration [days] (mean, SD)^a^	5.7 (3.9)	6.3^*^ (3.3)	6.1^*^ (3.5)
Surgical duration [min] (mean, SD)^b^	44 (14)	48.7^*^ (15.4)	47.1^*^ (15.2)
Estimated Blood lose [ml] (mean, SD)^c^	51 (47)	46.0^*^ (45.5)	47.7^*^(46)
Follow-up duration [months] (mean, range)^c^	6.9 (3–27)	9.2^*^ (0–24)	8.3^*^ (0–27)
Reoperation			
Total	9/102 (8.8%)	26/278 (9.4%)	39/380 (9.2%)
Cut out	5	10	14
Hardware removal	0	3	8
Nonunion	1	7	8
AVN	3	6	10
AVN time of diagnosis from initial surgery	Case 1: 12 months Case 2: 7 months Case 3: 6 months		

^*^Weighted mean

^a^Data was found in 8 studies

^b^Data was found in 9 studies

^c^Data was found in 5 studies

Results comparing patients who underwent revision surgery (9) to all other patients (93) are presented in Table 2. Significant differences between the two groups were patient age, surgeon seniority and fixation quality assessed by radiographic measurements of the Lateral Parker ratio. No correlation to fracture stability was found.

**Table 2:** Risk factors for reoperation

	No revision	Revision	*P* value
Number of patients	93	9	
Average age [years] (range)	62.0 (18–95)	73.3 (58–82)	0.003
Gender			
Male	48	5	0.821
Female	45	4	
Fracture classification			
Gardens 1–2	66	7	0.665
Gardens 3–4	27	2	
Senior surgeon (%)	86	4	< 0.001
Admission to surgery			
< 24 h	44	5	0.641
24–48 h	41	4	
> 48 h	8	0	
Average hospitalising duration [days] (range)	5.8 (1–28)	4.9 (2–8)	0.567
Surgical duration [min] (mean, range)	43.9 (21–95)	51.1 (22–90)	0.402
Follow-up duration [months] (mean, range)	7.0 (5–28)	6.4 (1–14)	0.829
Estimated blood loss [ml] (mean, range)	53.5 (0–200)	35.6 (0–100)	0.288
BMI (mean, range)	25.7 (17.5–34.6)	24.4 (19.6–29.7)	0.381
Reported comorbidities [number of patients]			
Diabetes	24	2	0.118
Cardiovascular	30	8	
Neurological	8	0	
Chronic lung disease	4	0	
Active malignancy	14	0	
Radiographic measurements			
TAD	20.9 + − 5.7	19.7 + − 6.4	0.529
Parker ratio Ap*	43.5 + − 7.7	46.5 + − 7.8	0.277
Parker ratio Lat*	47.4 + − 6.7	54.7 + − 6.3	0.003

^*^Median ratio of screw from centre

Literature review revealed 16 articles which provided data on FNS-treated patients, however, six were excluded (two were written in Chinese [19, 20], three were biomechanical articles [4, 5, 21] and one was a technical note [22]). From the ten eligible articles [6–15], data on 278 patients treated with FNS was collected and included in the final analysis. Table 3 summarises the number of patients, inclusion criteria, follow-up period, complications, re-operations rate and the main conclusions of the included articles. Data on demographics, fracture classification, surgical time, estimated blood loss, initial hospitalisation duration, follow-up duration and patient-reported comorbidities of the 278 patients described in the literature is presented in Table 1. In addition, cumulative analysis on the above variables for all 380 patients treated with FNS (102 patients described in this study and 278 from the literature review), is presented in Table 1. Finally, data regarding postoperative complications requiring revision surgery from the literature review and the 102 patients included in this paper is summarised and presented in Table 1.

**Table 3:** Literature review

Authors/year	Origin	FNS patients	Comparison group/number of patients	Inclusion criteria	Follow-up (months)	Additional data collected	Number of reoperations (group/cause)	Results/conclusions FNS group
Vazquez et al./04.08.2021	Switzerland	15	DHS-16 CS-32	Patient’s age > 75 Fractures classified: Gardens 1 and 2, posterior tilt < 20°	30 days	Radiographic femoral neck shortening	CS-1	• Shorter operative time • Reduced radiographic secondary fracture displacement
Cintean et al. / 19.9.2021	Germany	29	HA-34	Fractures classified: Garden 1 ASA score > 3	0–22	Charite Mobility Index (CHARMI)	FNS-4 (all cutout) HA-3 (1 dislocation, 2 infections)	• Lower nonsurgical complications • Shorter hospital stay, lower mortality rate
Nibe et al./ 30.04.2021	Japan	25	Other-27	Patient age > 65	> 6		**non** FNS group 6 (4 cut-outs, 2 hardware removals)	• Shorter surgical time • Lower reoperation rate
Tang et al./16.08.2021	China	47	CS-45	> 12-month follow-up	14–24	Radiographic femoral neck shortening Fracture healing time Harris hip score	FNS group 6 (1 AVN, 3 cut-outs, 2 nonunions) CS group 12 (3 AVN, 5 cut-outs, 4 nonunions)	• Lower radiographic femoral shortening • Shorter fracture healing time • Better Harris hip score at last follow-up
Stassen et al./ 07.11.21	Germany	34	No comparison	All nondisplaced FNF Displaced FNF for patients aged < 65	> 12	Fracture healing time	FNS 8 (4 AVN, 2 cut-outs, 2 irritations from hardware)	• Failure rates and technical difficulty are at least comparable to be established • CRIF systems
Niemann et al./26.02.2022	Germany	12	DHS-19	FNF	> 3	Radiation exposure	Non reported	• Lower radiation exposure • Shorter operative time
Zhang et al./18.02.2022	China	33	CS-36	Age < 65	> 6	Radiation exposure Harris hip score	CS-5 hardware removals	• Improved surgical time • Lower radiation • Better Harris hip score • Lower removal rate
Zhou et al./13.08.2021	China	30	CS-51	Age < 65	10–22	Harris hip score Time to walk Cost analysis	FNS 1–refracture CS-4 (2 nonunions, 3 hardware removals, 1 AVN)	• Decreased reoperation • Higher cost • Higher intraoperative bleeding
He et al. / 29.11.2021	China	33	CS-29	Age 18–65	12–24	Radiation exposure fracture healing time Harris hip score	FNS 4 (2 nonunions, 1 cut-out, 1 hardware removal) CS 8 (2 cut-outs, 2 nonunions, 2 hardware removals, 2 other)	• No significant differences in fracture healing • Time and hip function • Reduced radiation exposure • Lower number of postoperative complications
Hu et al./09.07.2021	China	20	CS-24	Age < 65	12 (not clear)	Fracture healing time Radiographic femoral neck shortening	FNS 3 (2 nonunions, 1 AVN) CS 12 (6 cut-outs, 3 AVN, 3 nonunions)	• Lower incidence of femoral neck shortening and screw cut-out • Shorter operative time

*CS* cannulated screws, *HA* hip arthroplasty, *DHS* dynamic hip screw

Radiographs and descriptions of three different patients from this study cohort are presented in Figs. 1, 2, 3, 4, 5, 6, 7, 8, 9.

**Fig. 1:**
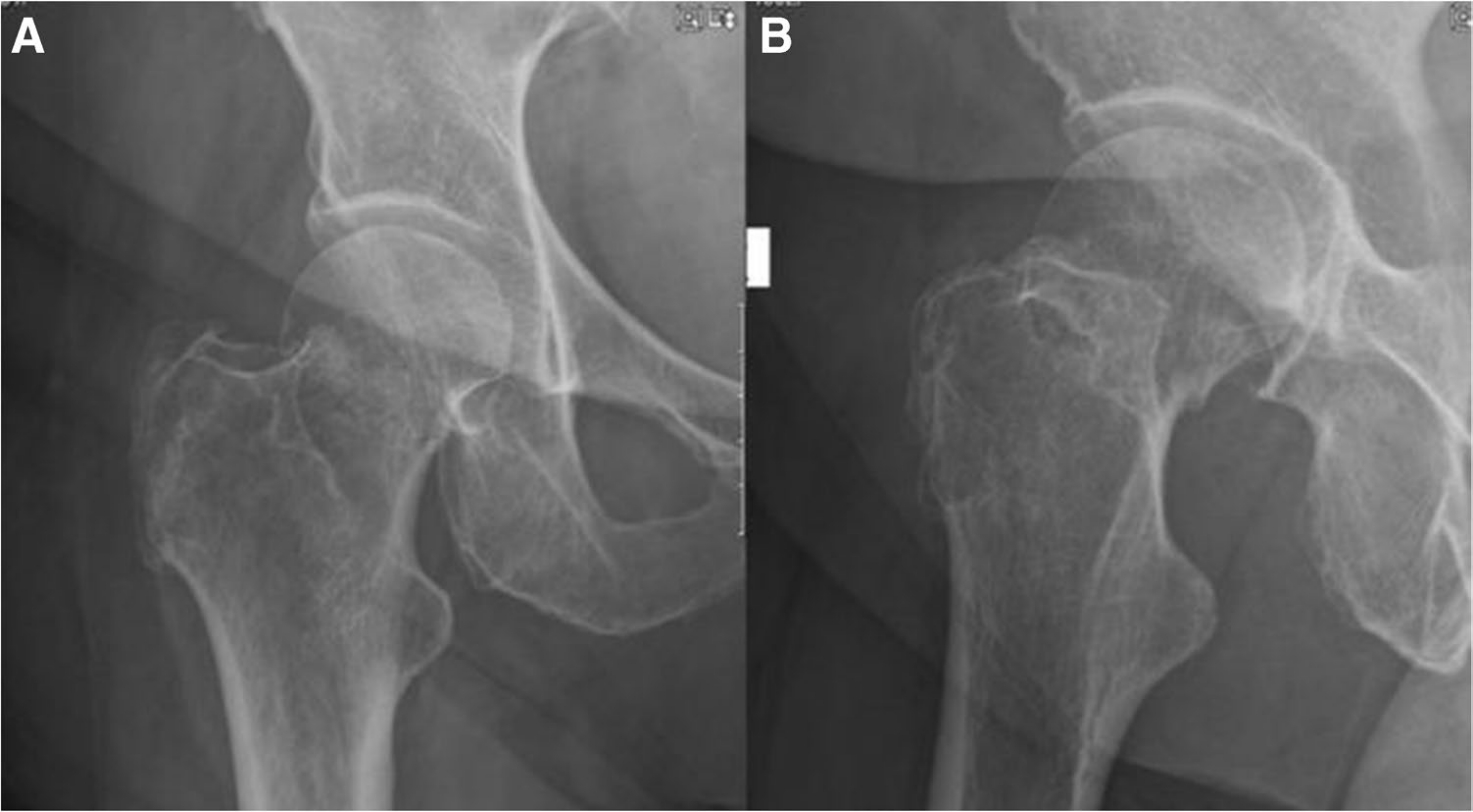
**A**, **B** AP and lateral radiographs showing intracapsular femur fracture of a 77-year-old female, which was admitted after a fall from standing height. Patient walks with no aids before the injury; her medical history consist of hypertension and dyslipidaemia

**Fig. 2:**
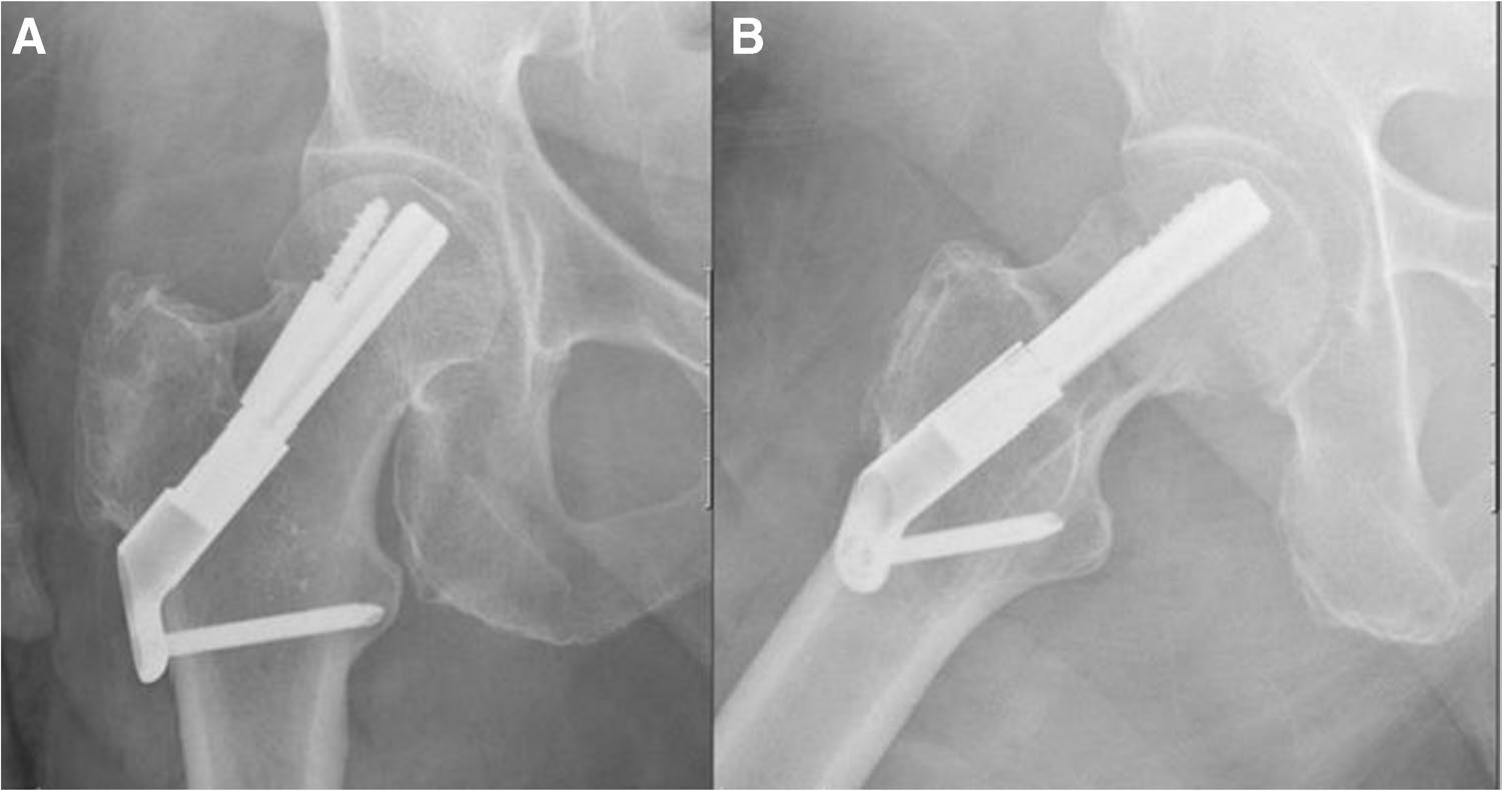
**A**, **B** AP and lateral radiographs postoperative day 1, demonstrating fixation of the fracture with FNS

**Fig. 3:**
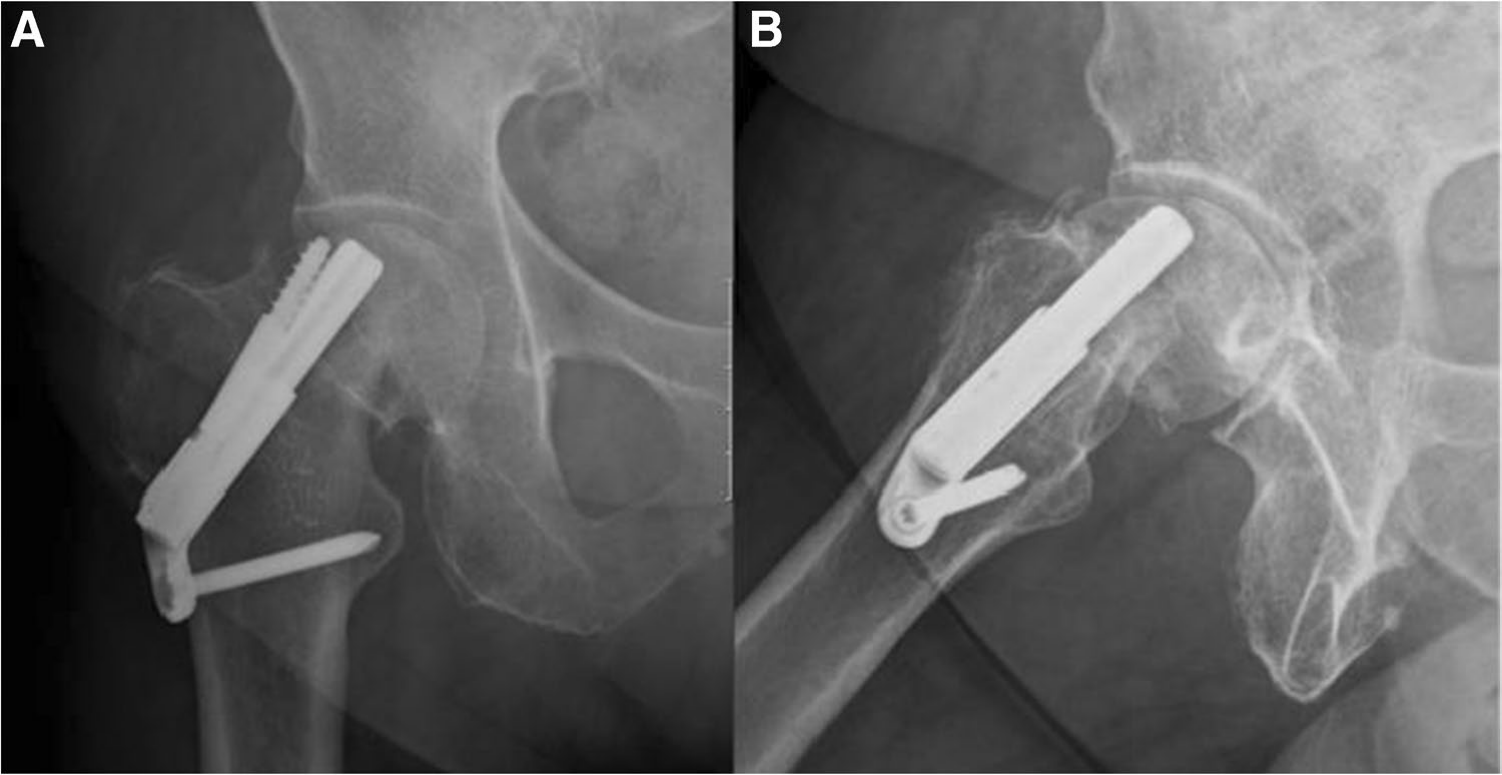
**A**, **B** AP and lateral radiographs 6 weeks after the surgical treatment. Radiographs demonstrate failure of fixation, cut-out of the implant. After the initial surgery, the patient was discharged home with instruction to full weight bear on the operated leg and was referred for physiotherapy treatment. Patient suffered from progressive hip pain and limp, walked short distance with the aid of a Zimmer frame. She was treated with revision surgery, hemiarthroplasty of the right hip

**Fig. 4:**
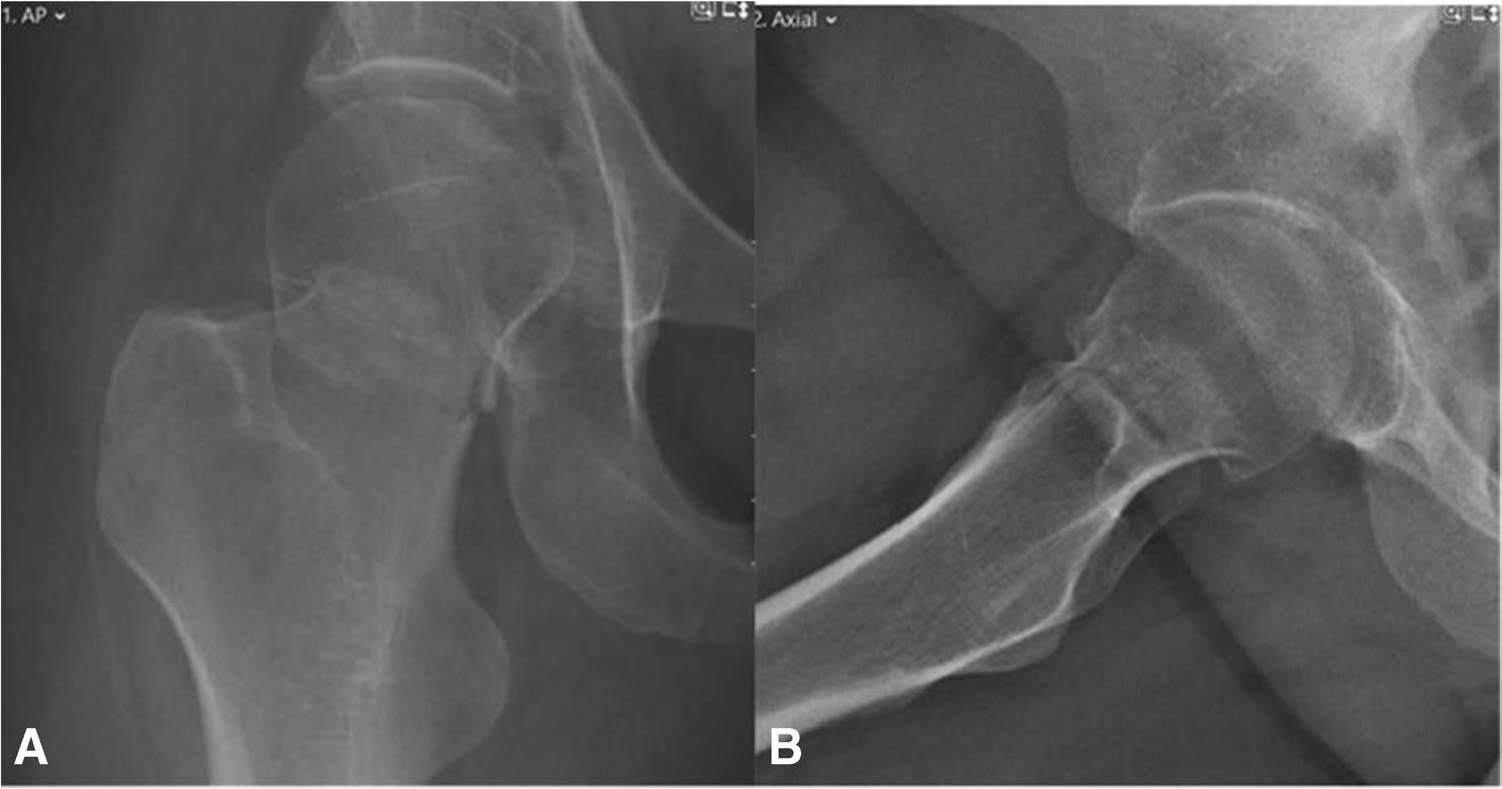
**A**, **B** AP and lateral radiographs showing intracapsular femur fracture (displaced, Garden 4) of a 39-year-old female. Past medical history consists of cerebral palsy, walks with a cane. Sustained an isolated injury to the right hip after falling from standing height

**Fig. 5:**
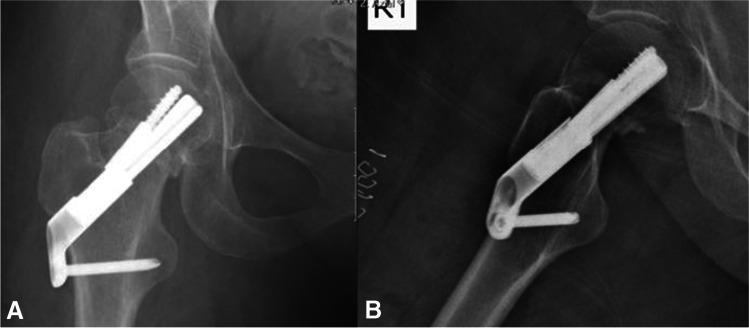
**A**, **B** AP and lateral radiographs post-operative day 1, demonstrating fixation of the fracture with FNS

**Fig. 6:**
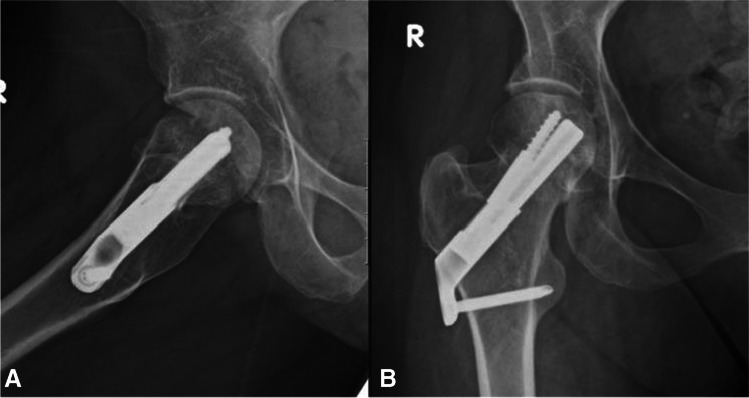
**A**, **B** AP and lateral radiographs from last follow-up 27 months after surgical treatment. Returned to her preinjury mobility status

**Fig. 7:**
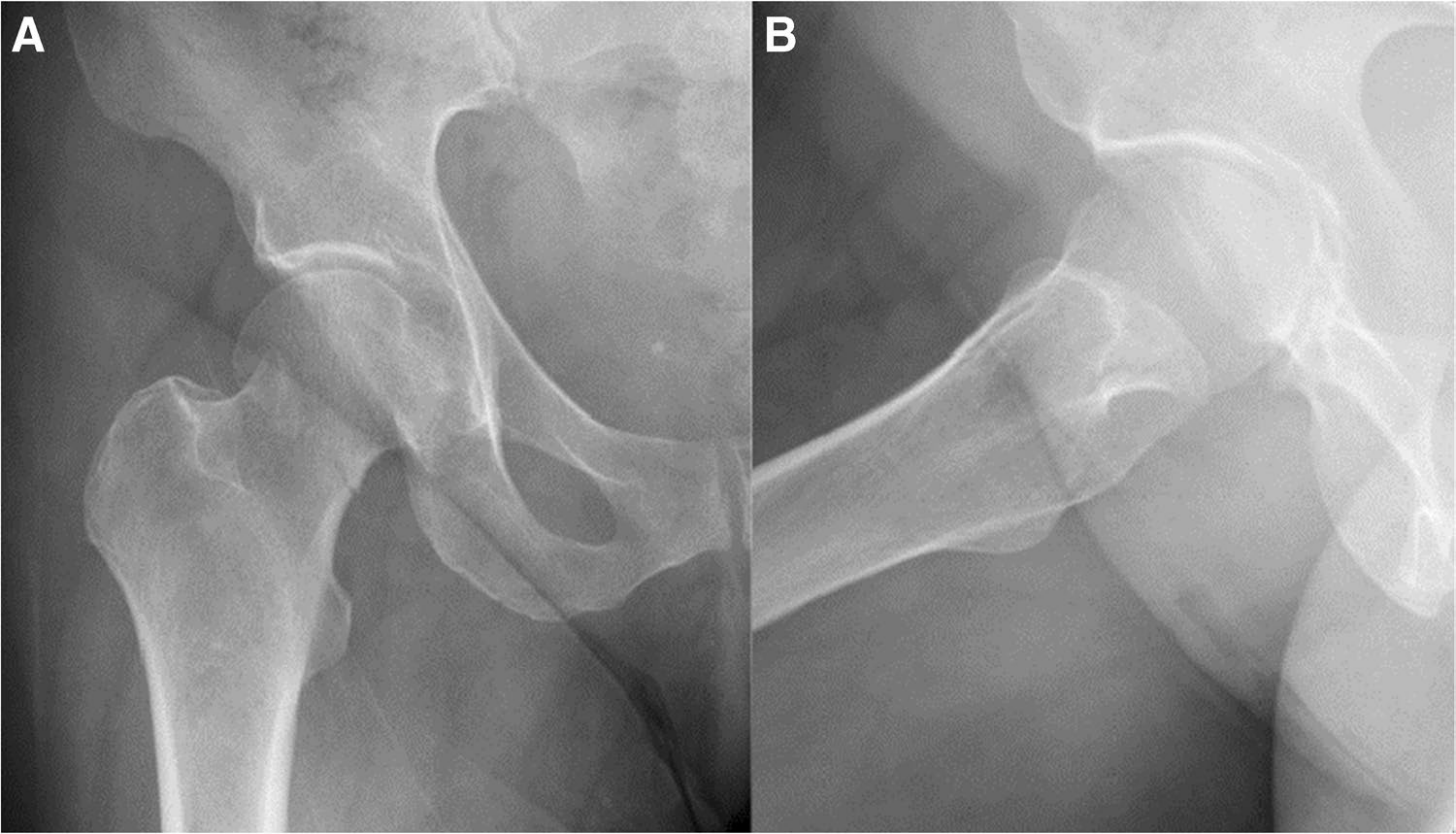
**A**, **B** AP and lateral radiographs showing right intracapsular femur fracture of a 71-year-old female. An isolated injury to the right hip after falling from own height when getting out of bed. Past medical history consists of chronic lymphocytic leukaemia, walks unaided

**Fig. 8:**
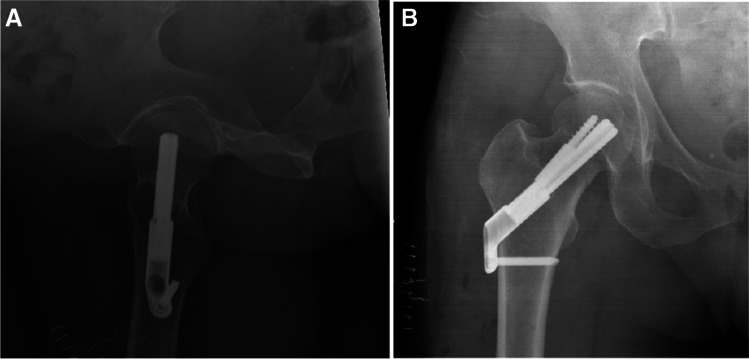
**A**, **B** AP and lateral radiographs postoperative day 1, demonstrating fixation of the fracture with FNS

**Fig. 9:**
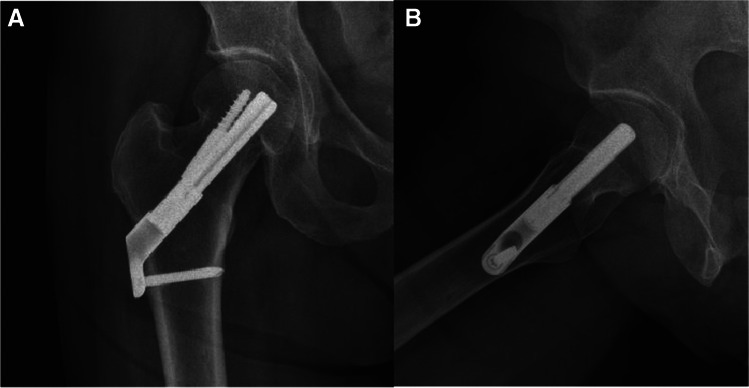
**A**, **B** AP and lateral radiographs from last follow-up, 24 months after the surgical treatment. Walks unaided, no complaints of chronic hip pain

## Discussion

In this study, we present data regarding 102 patients treated with FNS, and this represents the largest number of patients reported in literature to date. Adding data from previous publications enable us to analyse 380 patients treated with FNS. This study shows that FNS is a safe treatment option for FNF. Intraoperative parameters and clinical outcome of patients treated with FNS are comparable to those observed in other frequently used implants for fixation of FNF [23, 24].

Reported reoperation rates of FNF vary; 8% for non-displaced fractures and up to 42% in displaced fractures have been reported [25–27]. In the FAITH study [24], a large randomised control study of FNF treated by different fixation constructs, the reoperation rate was 21%. Re-operation rate for all patients treated with FNS analysed in this study was 9.2%. In the literature review, one study showed reduced reoperation rate in the FNS group in comparison to the alternative implant [9]; in contrast, all other studies showed similar re-operation rates. The average follow-up period for this study was  seven months (range 3–27). The relatively short follow-up period can be explained by the fact that the FNS was released only in 30 January 2019, and since then, it has been gradually used in our institutes. Generally, the majority of the reoperations of FNF occur in the first 12 months, in particular, re-operations related to fracture nonunion. The low number of nonunions, 1% (1/102), shown in our cohort, is an important finding and might reflect the biomechanical advantages of this implant. Longer follow-up would be ideal for exploring reoperation rates of FNFs. However, we believe that providing failure rates of a new implant, even before the desirable follow-up duration, can still provide useful information to both patients and clinicians.

Our study investigated intraoperative parameters of patients treated with FNS. The average operative time was 44 (range 21–95) minutes. This operative time is not dissimilar to other reported operative times for similar implants such as the Targon FN being 56 minutes [23]. Interestingly, three studies showed significant shorter operative time in the FNS group compared to the alternative implant [8, 9, 12]. One might have expected that a new implant would require longer operative time. The reported short operative time can be attributed to the low number of surgical steps in FNS which enhances procedural efficiency.

The average estimate blood loss for the FNS procedure was 47 (ml), an estimate, which is in the lower reported range for similar procedures. Fox et al. reported median intra-operative blood loss of 50 (ml) for CS and 200 (ml) for HA and DHS [28]. Other intra/peri-operative parameters which were reported for the FNS are reduced radiation exposure [7, 14, 15], shorter operation time [8, 9, 12, 14, 15], reduced initial hospitalisation and nonsurgical complication rate such as urinary tract infection and pneumonia [6, 7], reduced femoral neck shortening [8, 11, 14] and improved functional outcome (Harris hip score) [11, 14]. All these parameters demonstrate that the FNS is a relatively minimally invasive and operator-friendly implant.

Several predisposing factors are related to fixation failure in FNF, including female sex, increased BMI, older age, fracture type and suboptimal fracture reduction and implant positioning [24]. One of the objectives of this study was to identify risk factors for re-operation in patients treated with FNS. Our study found that patient’s age, surgeons’ seniority and precise surgical placement of implants were factors which affected the rate of re-operations. Only one other study evaluated risk factors for failure in patients treated with FNS and found patient age and presence of chronic lung disease as risk factors [10].

One of the limitations of this study is the short follow-up period. A longer follow-up period is necessary to effectively evaluate outcomes of FNF, preferably at 24 months. In addition, a larger cohort would enable assessment of the different fracture’s subgroups, displaced and non-displaced, and different patients age groups. A multi-centre randomised control study, with a long follow-up period, would be desirable to provide solid conclusions regarding the potential superiority of this implant in relation to the alternative treatment choices.

In conclusion, our hypothesis that the FNS implant would demonstrate analogous results to similar implants used for fixation of FNFs was confirmed. This study supports the view that FNS is a safe treatment option for FNF as shown in previously published literature.

## Data Availability

Raw data were generated from electronical records of the participating medical centres. Derived data supporting the findings of this study are available from the corresponding author [A.D] on request.
